# Association of nasal septal deviation with the incidence of anxiety, depression, and migraine: A national population-based study

**DOI:** 10.1371/journal.pone.0259468

**Published:** 2021-11-04

**Authors:** Ki-Il Lee, Seung Min In, Jong-Yeup Kim, Jee-Young Hong, Kyung-Do Han, Jung-Soo Kim, Yong Gi Jung

**Affiliations:** 1 Department of Otorhinolaryngology-Head and Neck Surgery, Konyang University College of Medicine, Daejeon, Republic of Korea; 2 Myunggok Medical Research Institute, Konyang University College of Medicine, Daejeon, Republic of Korea; 3 Department of Biomedical Informatics, Konyang University College of Medicine, Daejeon, Republic of Korea; 4 Department of Statistics and Actuarial Science, Soongsil University, Seoul, Republic of Korea; 5 Department of Otorhinolaryngology-Head and Neck Surgery, Kyungpook National University Hospital, School of Medicine, Kyungpook National University, Daegu, Republic of Korea; 6 Department of Otorhinolaryngology-Head and Neck Surgery, Samsung Medical Center, Sungkyunkwan University School of Medicine, Seoul, Republic of Korea; Sapienza University of Rome, ITALY

## Abstract

**Background & aims:**

Nasal obstruction caused by nasal septal deviation is very bothersome and, therefore, can affect the patient’s emotional state. However, little is known about the effect of nasal septal deviation (NSD) on the neuropsychiatric aspects of patients. Therefore, this study aims to verify the higher incidence of anxiety, depression, and migraine in patients diagnosed with NSD compared to general populations using big data.

**Methods:**

This retrospective cohort study collected subjects from the Korean National Health Insurance Service (NHIS) database. Adjustments were made to minimize the confounding of variables for age, sex, residence type, income levels, hypertension, diabetes, dyslipidemia, rhinitis, and chronic rhinosinusitis between the two groups. The primary endpoint of this study was newly diagnosed anxiety, depression, and migraine between January 2009 and December 2018. Kaplan-Meier survival curves, logarithmic rank test, and Cox proportional regression test were used for statistical analysis.

**Results:**

Among a total of 135,769 subjects in the NHIS database, 48,495 patients with NSD (NSD group) and 54,475 control subjects (control group) were selected. Patients with NSD had an increased risk of anxiety, depression, and migraine compared to the control group. In the NSD group, the adjusted hazard ratios (HR) were 1.236 (95% CI, 1.198–1.276) for anxiety, 1.289 (95% CI, 1.238–1.343) for depression, and 1.251 (95% CI, 1.214–1.290) for migraine.

**Conclusion:**

NSD is associated with a higher incidence of anxiety, depression, and migraine. Therefore, it is suggested that physicians carefully consider psychoneurological distress and employ therapeutic strategies to minimize these conditions.

## Introduction

Nasal septal deviation (NSD) is one of the most prevalent upper airway diseases in an otorhinolaryngology clinic, causing nasal airway obstruction [1]. Generally, this common nasal pathology affects the quality of life (QOL) due to its rhinologic symptoms [2, 3]. Additionally, previous articles have reported non-rhinologic manifestations accompanying NSD, such as facial pain, sleep problems, and loss of productivity [4, 5]. Due to these effects on overall QOL, it can be assumed that NSD is also associated with neuropsychological disorders such as anxiety, depression, and migraine.

Previous articles have investigated the association between upper airway pathologies and neuropsychological symptoms [6, 7]. In addition, there were several retrospective cohort studies assessing depression and dementia in patients with obstructive sleep apnea (OSA) [8, 9]. On the contrary, there are only a few reports about NSD. Recently, Ma et al. [10] reported that depression and anxiety are more common and severe in patients with NSD through a case-control study. Meanwhile, Akkoca et al. [11] found elevated anxiety levels in patients with symptoms of nasal obstruction, which did not correlate with nasal endoscopic or radiological examinations. Folic et al. found a significant reduction in headache intensity and frequency in both groups of surgically treated patients (between the nasal septum and the inferior turbinate or the middle turbinate). However, 6 months after surgery, this reduction was more significant in patients with mucosal contact between the nasal septum and the middle turbinate [12].

Furthermore, for rhinogenic headaches, there is a debate on NSD and treatment [13, 14]. However, a recent meta-analysis confirmed that nasal surgery for rhinogenic contact point headache improves symptoms with both the visual analog scale and the assessment of migraine disability [15]. Furthermore, radiofrequency treatment of nasal turbinates demonstrated reasonable long-term pain control measured on subjective scales, which was correlated with restoration of mucociliary clearance and nasal health measured by nasal cytology [16]. These inconsistent results between NSD and neuropsychological disorders are caused by methodological limitations such as a small sample size, incomplete control of confounders, and incomplete analysis with only subjective questionnaires. Therefore, there is limited understanding of the effect of NSD on anxiety, depression, and migraine. Therefore, the assumption needs to be clarified in a large population with adjustment for risk factors in order to overcome methodological limitations.

Recently, the Korean National Health Insurance Service (NHIS) database has become available for medical research in South Korea. The NHIS includes a complete set of health data covering approximately 50 million of the South Korean population. Furthermore, the present cohort datasets from the NHIS database cover various information such as demographics, comorbidity, diagnostic codes with the International Classification of Disease-10 (ICD-10), and medical examination codes of procedures and prescriptions. Therefore, this study aimed to verify whether patients with NSD are at high risk for anxiety, depression, and migraine by analyzing large cohort data sets.

## Materials and methods

### Data source

This retrospective cohort study was performed using the NHIS claims database. NHIS is a government organization that manages a national insurance system in Korea, covering approximately more than 95% of the Korean population [8, 17, 18]. The cohort information involves all forms of medical service use such as the following: 1) personal demographics, 2) death, 3) prescription, 4) diagnosis, 5) procedure, 6) medical costs, 7) hospital or department, and 8) national health screening. In particular, diagnostic data are based on ICD-10 codes, and all hospital and out-patient claims data can be reviewed. In addition, national health screening information for the entire Korean population can be achieved because most individual subjects are recommended to undergo standardized medical examinations twice a year.

Furthermore, all people in Korea have a resident registration number, and the Korean insurance system covers most. All hospitals and private clinics are required to use this resident registration number before practicing. Therefore, the NHIS covers most Korean citizens and remains accurate even if a subject moved to a different regional area. This system was mainly intended for insurance claims and nationwide medical management, but recently the data became available for medical research. Therefore, numerous studies have emerged in Korea using the NHIS claims database.

### Study population and setting

Patients over 20 years of age with newly diagnosed NSD (J342) between January 1, 2009, and December 31, 2009, from the NHIS database, were designated as the NSD group. To improve diagnostic precision, as previously reported [19], only those who had undergone nasal endoscopy (E7530, E7540, E7550, or E7560) were enrolled. The exclusion criteria were as follows: 1) patients diagnosed with NSD before enrollment, 2) patients who underwent previous rhinologic surgeries such as septoplasty, turbinate reduction, rhinoplasty, or endoscopic sinus surgery, and 3) patients younger than 20 years or older than 80 years. Subjects who were not diagnosed with NSD were selected with matching according to age and sex using the NHIS, and this was designated as the control group. The washout of previously diagnosed anxiety (F41), depression (F32 or F33), and migraine (G43 or G44) was applied. Moreover, newly diagnosed anxiety, depression, and migraine for 10 years, from January 1, 2009, to December 31, 2018, were the outcomes of interest in our study (Table 1).

**Table 1:** Working definition derived from the NHIS claim database.

Disease	Working definition
Nasal septal deviation	At least one claim under ICD-10 codes J34.2 + nasal endoscopy (E7530, E7540, E7550 or E7560)
Anxiety	At least one claim under ICD-10 codes F41
Depression	At least one claim under ICD-10 F32 or F33
Migraine	At least one claim under ICD-10 G43 or G44
Diabetes	At least one claim under ICD-10 E11-14 + ≥ 2 out-patient visits or ≥ 1 admission within 1 year
Hypertension	At least one claim under ICD-10 I10-13 or I15 + ≥ 2 out-patient visits or ≥ 1 admission within 1 year
Dyslipidemia	E78 + ≥ 2 out-patient visits or ≥ 1 admission within 1 year
Rhinitis	J30 + ≥ 3 out-patient visits within 1 year
Chronic rhinosinusitis	J32 + ≥ 1 out-patient visit within 3 months + nasal endoscopy (E7530, E7540, E7550 or E7560)

Abbreviation: ICD-10, International Classification of Diseases, 10^th^ edition.

To improve adjustment, we obtained the following baseline characteristics from the NHIS database: age, gender, urban residency, and income level (lowest quintile). Furthermore, to control and minimize confounders, chronic comorbid medical diseases of patients such as hypertension (I10–I13 or I15 + ≥ 2 out-patient visits or ≥ 1 admission within 1 year), diabetes (E11–14 + ≥ 2 out-patient visits or ≥ 1 admission within 1 year) and dyslipidemia (E78 + ≥ 2 out-patient visits or ≥ 1 admission within 1 year), as well as common rhinological disorders such as rhinitis (J30 + ≥ 3 out-patient visits within 1 year) and chronic rhinosinusitis (CRS) (J32 + ≥ 1 out-patient visit within 3 months + nasal endoscopy) were collected (Table 1). These diagnostic definitions were followed or modified as described in previous studies [20–23]. Subsequently, a multivariate adjustment was performed for age, sex, income level, systemic comorbidity, and rhinological disease.

All four models were used for this retrospective cohort analysis. However, the first model was not adjusted, the second model was adjusted only for age and gender, the third model was complexly adjusted for age, gender, income level, and basic comorbidities (i.e., hypertension, diabetes, and dyslipidemia), and additionally, the fourth model included the adjustment for rhinitis and CRS.

### Ethical approval

This study was approved by the institutional review board of Konyang University Hospital, Daejeon, Republic of Korea (2020-01-013). However, informed consent could not be obtained because the anonymized national health database was used for analysis in this study.

### Statistical analysis

Variables are classified as frequencies and percentages. Data are presented as mean ± standard deviation for age and as proportions for each categorical variable. The crude incidence rates for anxiety, depression, and migraine were analyzed as the number of each event per 1000 person-years. Kaplan–Meier curves were used to calculate the cumulative incidence of these symptoms between the NSD and control groups. To examine the hazard ratio (HR) of NSD for the incidence of anxiety, depression, and migraine, the Cox proportional risk model was used to analyze adjustment models. HR was calculated with a 95% confidence interval (CI).

Statistical tests were performed using SAS Version 9.4 (SAS Institute, Inc., Cary, NC) and R version 4.0.3 (The R Foundation for Statistical Computing, Vienna, Austria). All analyzes were performed using a two-tailed 95% CI. *P* < 0.05 was considered statistically significant.

## Results

**Fig 1:**
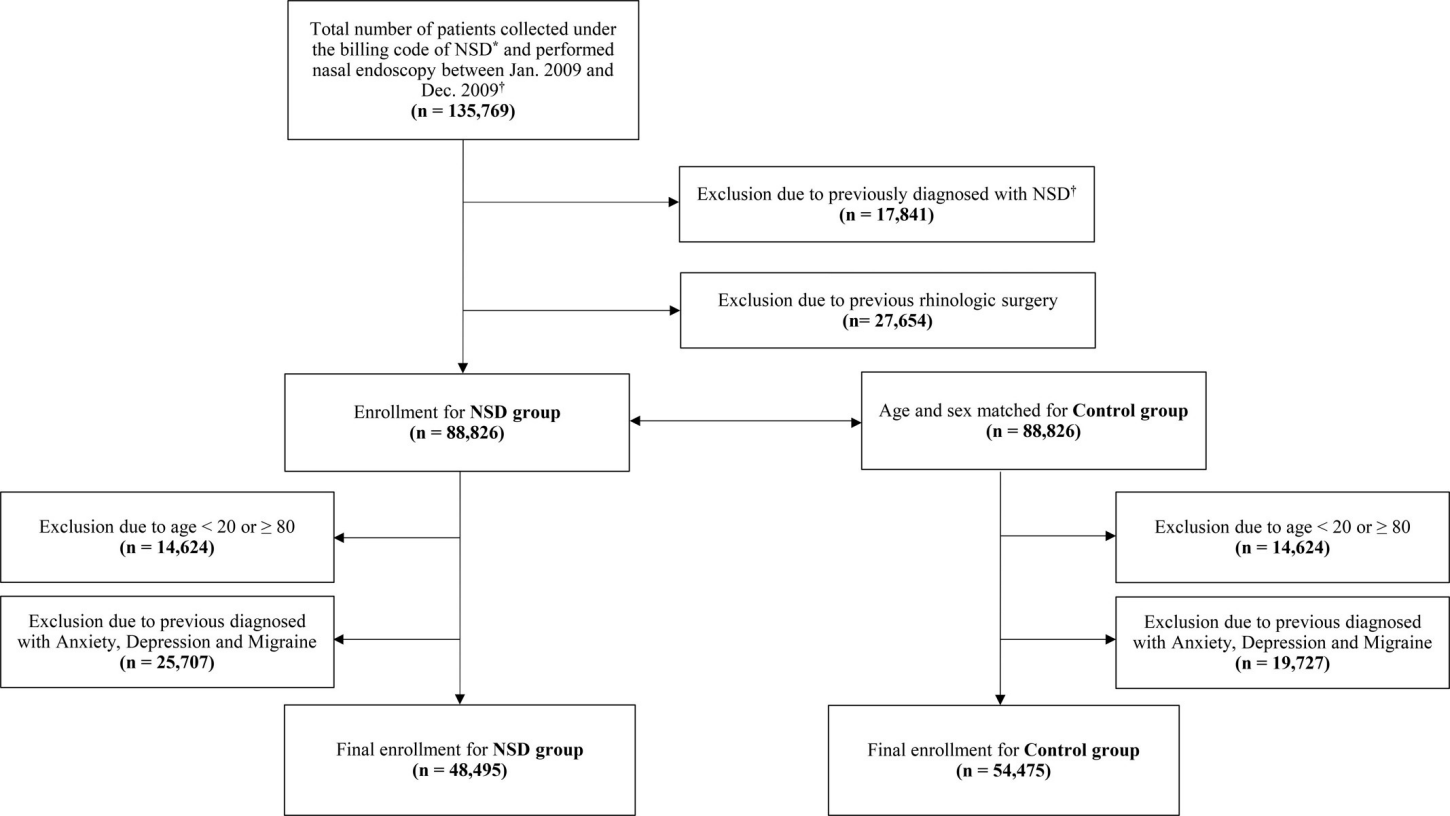
A schematic flow diagram of the subject selection in the present study. Of 135,769 patients diagnosed with NSD in 2009, 88,826 patients were selected. Participants with previous NSD and rhinologic surgery were excluded. NSD participants were matched with a control group who had not been diagnosed with NSD. Participants under 20 years of age or over 80 years of age and previous psychoneurological disorders were also excluded. Finally, 48,495 participants with NSD and 54,475 control participants were included.

Between January 1, 2009, and December 31, 2009, there were 135,769 patients newly diagnosed patients with NSD. Among these subjects, 48,495 patients with NSD were evaluated. As a control group, 54,475 subjects were collected from the general population (Fig 1). Their demographic data are shown in Table 2.

**Table 2:** Demographic characteristics of patients with NSD and the control group.

Variables	Control	NSD	*P*-value*
	(n = 54475)	(n = 48495)	
Male	36000 (66.1%)	32135 (66.3%)	0.54
Mean age (year)	38.26 ± 12.82	37.65 ± 12.54	< 0.001
Age ≥ 40 years	21602 (39.7%)	18139 (37.4%)	< 0.001
Income lowest quintile	9282 (17.0%)	6704 (13.8%)	< 0.001
Urban residency	33811 (48.7%)	33120 (45.7%)	< 0.001
Diabetes	1915 (3.5%)	1732 (3.6%)	0.63
Hypertension	3846 (7.1%)	3823 (7.9%)	< 0.001
Dyslipidemia	2110 (3.9%)	2478 (5.1%)	< 0.001
Rhinitis	3936 (7.2%)	14112 (29.1%)	< 0.001
CRS	761 (1.4%)	18101 (37.3%)	< 0.001

Data are expressed as counts (percentages) for categorical variables and mean ± standard deviations for continuous variables.

^*^P values were determined using the Student *t*-test for continuous variables and Pearson’s χ^2^ test for categorical variables.

Abbreviation: NSD, nasal septal deviation; CRS, chronic rhinosinusitis.

### Comparison of the incidence of anxiety between the NSD and control groups

**Fig 2:**
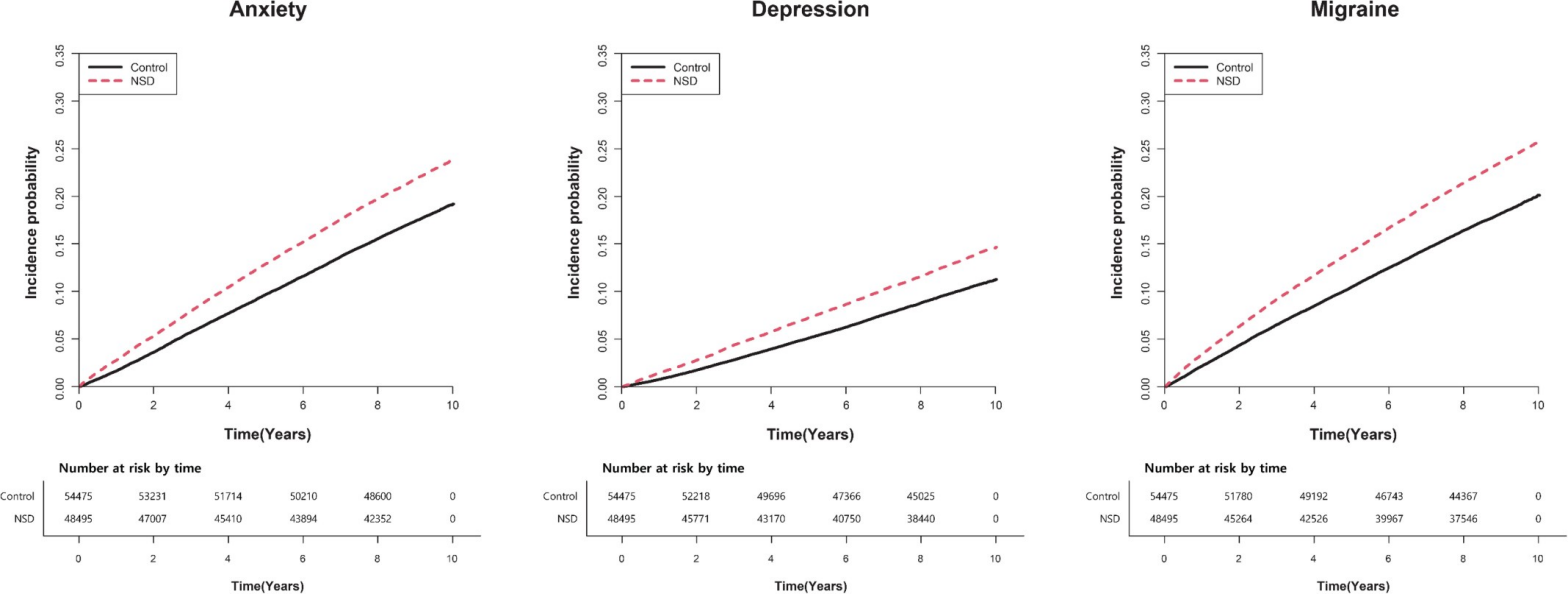
Kaplan–Meier curves of the incidence of anxiety, depression, and migraine in patients with NSD. Anxiety, depression, and migraine occur more frequently in the NSD group compared to the control group.

Anxiety occurred more frequently in the NSD group compared to the control group. The Kaplan–Meier plot revealed that anxiety was higher in the NSD group than in the control group over time (Fig 2). This outcome was seen in all four models. The incidence rates of anxiety were 21.221 and 27.358 per 1000 person-years in the control and NSD groups, respectively. The anxiety HR in the NSD group were 1.289 (1.255–1.325), 1.319 (1.284–1.355), 1.316 (1.281–1.353), and 1.236 (1.198–1.276) in models 1, 2, 3, and 4, respectively. Detailed results are presented in Table 3.

**Table 3:** Crude and adjusted hazard ratios of NSD for anxiety, depression, and migraine.

Variables	Groups	N	Events	Duration	Rate	Model 1	Model 2	Model 3	Mode 4
Anxiety	Control	54475	9855	464409.98	21.221	1	1	1	1
						(Reference)	(Reference)	(Reference)	(Reference)
	NSD	48495	11011	402477.33	27.358	1.289	1.319	1.316	1.236
						(1.255–1.325)	(1.284–1.355)	(1.281–1.353)	(1.198–1.276)
Depression	Control	54475	5738	485587.45	11.817	1	1	1	1
						(Reference)	(Reference)	(Reference)	(Reference)
	NSD	48495	6721	426085.57	15.774	1.336	1.366	1.367	1.289
						(1.290–1.384)	(1.319–1.415)	(1.319–1.416)	(1.238–1.343)
Migraine	Control	54475	10303	459677.95	22.414	1	1	1	1
						(Reference)	(Reference)	(Reference)	(Reference)
	NSD	48495	11893	396331.41	30.008	1.338	1.345	1.345	1.251
						(1.303–1.374)	(1.310–1.381)	(1.310–1.381)	(1.214–1.290)

Data are expressed as the hazard ratio (95% confidence interval). Model 1 was non-adjusted; Model 2 was adjusted by age and sex; Model 3 was adjusted by age, sex, income level, diabetes, hypertension, and dyslipidemia; and Model 4: adjusted by age, sex, income level, diabetes, hypertension, dyslipidemia, rhinitis, and chronic rhinosinusitis.

Abbreviation: NSD, nasal septal deviation.

### Comparison of incidence of depression between the NSD and control groups

Depression was more frequent in the NSD group than in the control group (Table 3). In addition, the Kaplan–Meier curve of the NSD group showed a higher incidence of depression over time than that of the control group (Fig 2). This outcome was also shown in adjusted models (models 2, 3, and 4). The incidence rates of depression were 11.817 and 15.774 per 1000 person-years in the control and NSD groups, respectively. The HR of depression in the NSD group was 1.336 (1.290–1.384), 1.366 (1.319–1.415), 1.367 (1.319–1.416), and 1.289 (1.238–1.343) in models 1, 2, 3, and 4, respectively.

### Comparison of the incidence of migraine between the NSD and control groups

The HR of migraine was higher in subjects with NSD compared to control subjects in all models. After calculating the change over time, the gradual increase in the incidence of migraine was shown in the NSD, and control groups were shown (Fig 2). The incidence rates for migraine were 22.414 and 30.008 per 1000 person-years in the control and NSD groups, respectively. The HR of migraine in the NSD group were 1.338 (1.303–1.374), 1.345 (1.310–1.381), 1.345 (1.310–1.381), and 1.251 (1.214–1.290) in models 1, 2, 3, and 4, respectively. The results are presented in Table 3.

### Incidence of anxiety, depression, and migraine among subjects with NSD according to several covariates

According to several covariates, the incidences of anxiety, depression, and migraine were different as follows (anxiety, depression, and migraine, respectively): 1.133 (1.078–1.191), 1.270 (1.191–1.354), in women, respectively; 1.217 (1.164–1.272), 1.217 (1.164–1.272), 1.225 (1.168–1.286) in ≥ 40 years of age, respectively; 1.088 (0.968–1.223), 1.217 (1.053–1.407), 1.171 (1.026–1.338) in hypertension, respectively; 1.048 (0.977–1.123), 1.215 (0.996–1.483), 1.006 (0.874–1.157) in CRS, respectively. Interestingly, the HR values of anxiety, depression, and migraine were lower for subjects older than 40 years. Additionally, the HR values of anxiety, depression, and migraine were attenuated in women and those with comorbidities such as diabetes, hypertension, rhinitis, and CRS compared to subjects in the control group with the same condition. On the contrary, the HR value of depression (1.345 [1.203–1.503]) was not lower for subjects with dyslipidemia than for comparison subjects (1.278 [1.223–1.335]). Detailed results are presented in Table 4.

**Table 4:** Hazard ratios (95% CI) of various covariates on the incidence of anxiety, depression, and migraine in patients with NSD.

Variables		Anxiety		Depression		Migraine	
		Control	NSD	Control	NSD	Control	NSD
Sex	Male	1 (Reference)	1.312 (1.260–1.367)	1 (Reference)	1.304 (1.237–1.374)	1 (Reference)	1.346 (1.293–1.401)
	Female	1 (Reference)	1.133 (1.078–1.191)	1 (Reference)	1.270 (1.191–1.354)	1 (Reference)	1.132 (1.080–1.186)
Age	< 40	1 (Reference)	1.255 (1.200–1.312)	1 (Reference)	1.341 (1.264–1.422)	1 (Reference)	1.268 (1.219–1.318)
	≥ 40	1 (Reference)	1.217 (1.164–1.272)	1 (Reference)	1.243 (1.175–1.316)	1 (Reference)	1.225 (1.168–1.286)
Diabetes	No	1 (Reference)	1.238 (1.199–1.279)	1 (Reference)	1.287 (1.234–1.343)	1 (Reference)	1.252 (1.214–1.291)
	Yes	1 (Reference)	1.182 (1.036–1.348)	1 (Reference)	1.283 (1.098–1.500)	1 (Reference)	1.209 (1.030–1.418)
Hypertension	No	1 (Reference)	1.247 (1.207–1.288)	1 (Reference)	1.291 (1.237–1.347)	1 (Reference)	1.253 (1.215–1.293)
	Yes	1 (Reference)	1.088 (0.968–1.223)	1 (Reference)	1.217 (1.053–1.407)	1 (Reference)	1.171 (1.026–1.338)
Dyslipidemia	No	1 (Reference)	1.236 (1.196–1.279)	1 (Reference)	1.278 (1.223–1.335)	1 (Reference)	1.257 (1.217–1.297)
	Yes	1 (Reference)	1.220 (1.116–1.334)	1 (Reference)	1.345 (1.203–1.503)	1 (Reference)	1.180 (1.061–1.313)
Rhinitis	No	1 (Reference)	1.267 (1.223–1.313)	1 (Reference)	1.308 (1.249–1.368)	1 (Reference)	1.291 (1.248–1.336)
	Yes	1 (Reference)	1.085 (1.009–1.167)	1 (Reference)	1.172 (1.065–1.290)	1 (Reference)	1.048 (0.977–1.123)
CRS	No	1 (Reference)	1.243 (1.203–1.284)	1 (Reference)	1.287 (1.234–1.343)	1 (Reference)	1.254 (1.215–1.294)
	Yes	1 (Reference)	1.043 (0.901–1.206)	1 (Reference)	1.215 (0.996–1.483)	1 (Reference)	1.006 (0.874–1.157)

Data are expressed as hazard ratio (95% confidence interval). Model 1 was non-adjusted; Model 2 was adjusted by age and sex; Model 3 was adjusted by age, sex, income level, diabetes, hypertension, and dyslipidemia; and Model 4: adjusted by age, sex, income level, diabetes, hypertension, dyslipidemia, rhinitis, and chronic rhinosinusitis.

Abbreviations: NSD, nasal septal deviation; CRS, chronic rhinosinusitis.

## Discussion

To our knowledge, this study is the first nationwide analysis to investigate the risk of anxiety, depression, and migraine in patients with NSD using big data. In this study, the incidence of neuropsychological disorders among patients with NSD was significantly higher than that in the control group. Furthermore, even after adjusting for demographic findings, chronic systemic diseases, and even rhinological illnesses, HR for anxiety, depression, and migraine remained significantly higher in patients with NSD compared to controls.

Currently, little is known about the effect of NSD on affective disorders such as anxiety and depression. Preliminarily, Fidan et al. [24] reported that psychiatric symptoms such as somatization, obsession, depression, anxiety, and even psychoticism are common in patients with NSD. In their study, data were collected via preoperative and postoperative questionnaires among 40 patients with NSD and 36 healthy volunteers. They also asserted that nasal obstruction aggravates a decrease in QOL and an impairment in sleep quality. Surgical correction provides a more effective result in patients with rhinogenic headache, although it is difficult to determine, especially in the presence of an isolated mucosal contact point. For this reason, medical treatment should initially be used as a guide for a future surgical indication [25, 26]. The present study validated the association of NSD with anxiety and depression after analyzing long-term follow-up NHIS data based on the national population of approximately 50 million people. Regarding migraine, previous studies revealed that anatomic variations in the sinonasal area (i.e., NSD, concha bullosa, Haller cell, and Onodi cell) might cause rhinogenic contact headache or migraine [27, 28]. Clinically, numerous patients have suffered from rhinogenic headache in the absence of neurologic pathology caused by NSD [2, 13]. Recently, Kwon et al. [19] reported the association of NSD with headache in a nationwide cohort study in Korea. Our adjusted HR for migraine (1.345; 95% CI, 1.310–1.381) was similar to the study by Kwon (1.37; 95% CI, 1.31–1.43) under the same condition. However, our study has several strengths compared to their study. First, we analyzed a customized dataset from the entire national population of Korea (approximately 50 million people) instead of using a representative national sample dataset of 1 million people. Second, our analysis involved an adjusted model accounting for rhinological disorders (e.g., rhinitis and CRS) and chronic systemic diseases (e.g., diabetes, hypertension, and dyslipidemia). Third, we also performed HR analyses for migraine and affective disorders such as anxiety and depression.

The mechanism by which NSD contributes to these psychoneurological disorders is thought to be multifactorial. There are several possible explanations for the relationship between NSD and psychoneurological disorders. First, anatomically, NSD causes a continuous, direct stimulus to the head and neck area. In addition to being a breathing problem, this could also affect the QOL of patients by causing concentration difficulties, sleep problems, and even mood changes [4]. A sense of nasal blockage caused by NSD can cause negative feelings or continuous mood changes, leading to a vicious cycle of anxiety, depression, and migraine. Second, histologically, NSD could be associated with microscopic changes in the nasal mucosa. In previous studies, histopathological changes, such as impaired mucociliary transport and accompanying mucosal inflammation, have been associated with NSD [29, 30]. The relationship between airway inflammation and neurological function, cognition, and a sense of well-being was demonstrated in previous studies [31, 32]. Third, immunologically, NSD can continuously invoke chronic mucosal inflammation by releasing inflammatory cytokines into the peripheral airway mucosa [29]. Local inflammatory reactions and released cytokines may induce neuropsychiatric disorders [33]. Fourth, physically, patients with NSD may suffer from decreased activity, which is associated with obesity and other systemic comorbidities. Obesity and decreased physical activity are well-known risk factors for anxiety, depression, and migraine [34, 35].

Our study also demonstrated the effects of several covariates, including sex, age, diabetes, hypertension, dyslipidemia, rhinitis, and CRS. There was a difference in HR between the NSD and comparison groups according to age, gender, and comorbidities. Sex-based discrepancies have been reported in patients with upper airway obstruction, and this could be due to the existence of anatomical, hormonal, and endocrinological differences [36]. In the present study, in patients with NSD, higher HR was found for anxiety, depression, and migraine in men. Generally, although women are at increased risk for anxiety, depression, and migraine [37], the greater vulnerability of women to these diseases could vary with NSD.

Interestingly, younger patients with NSD showed an increased risk of depression, anxiety, and migraine compared to older people. This could be because older patients, who have had long-lasting blockage of the upper airways, have become more tolerant of subjective affective symptoms than younger patients. This result could be in conjunction with a previous study on the age-related difference in OSA [38, 39]. Furthermore, in our study, patients with the chronic systemic disease might have a higher tolerance to NSD than those without comorbidities such as diabetes, dyslipidemia, and hypertension. NSD patients without systemic diseases had substantially higher HR except for dyslipidemia for depression than those with chronic diseases. This may be because patients diagnosed with more comorbid diseases receive more medical services. In the present study, rhinologic disorders such as rhinitis and sinusitis were more prevalent in patients with NSD than in the control group. Compared to other patients, in addition, subjects without these prevalent rhinologic disorders were at greater risk for neuropsychological disorders. This outcome verifies that having an NSD increases the risk of depression, anxiety, and migraine, which ruled out the effect of other rhinological illnesses.

In contrast, this study still has some limitations. First, the severity of the disease could not be evaluated. Most previous studies on NSD could not be classified according to the severity of NSD, even randomized controlled studies [2, 10]. This is because most NSDs are diagnosed based on clinical judgment, which is a common limitation in studies on NSD. Second, the subtypes of anxiety, depression, and migraine were not considered in defining the diagnosis. This is important because the clinical progression and prognosis of these neuropsychological diseases can vary. For example, there might be clinical discrepancies between simple anxious depression and major depressive disorder. In the future, a large prospective study using hospital data is needed to overcome these limitations and validate our findings.

## Conclusion

By analyzing the NHIS claim database, we found that there is a higher incidence of anxiety, depression, and migraine in patients with NSD than the general population. Clinically, these findings should be considered during the diagnostic and therapeutic process to improve the QOL of patients with NSD.

## Supporting information

S1 Table Minimal data set underlying the results described in the manuscript. (XLSX)
